# Long-Term Patient Satisfaction and Oral Health-Related Quality of Life Following Anterior Maxillary Implant Placement: A Five-Year Follow-Up Study

**DOI:** 10.1055/s-0045-1811195

**Published:** 2025-08-13

**Authors:** Hanen Boukhris, Hajer Zidani, Ghada Bouslama, Kawther Bel Haj Salah, Souha BenYoussef

**Affiliations:** 1 Department of Prosthodontics, Faculty of Dental Medicine, LR12SP10 University Hospital of Farhat Hached, University of Sousse, Sousse, Gouvernorat de Sousse, Tunisia; 2 Department of Oral Surgery, Faculty of Dental Medicine, LR12SP10 University Hospital of Farhat Hached, University of Sousse, Sousse, Sousse Médina, Gouvernorat de Sousse, Tunisia; 3 Department of Restorative Dentistry and Endodontics, Faculty of Dental Medicine, University of Monastir, Monastir, Tunisia

**Keywords:** anterior maxillary implants, five-year evaluation, implant-related quality of life, long-term follow-up, patient satisfaction

## Abstract

**Objectives:**

Long-term data evaluating both patient satisfaction and oral health-related quality of life (OHRQoL) in anterior maxillary implant rehabilitation are still limited, making this investigation highly relevant. This study aimed to evaluate patient satisfaction and OHRQoL 5 years after anterior maxillary implant placement. It specifically assessed long-term satisfaction with implant-supported restorations in the aesthetic zone, the impact on OHRQoL, and key clinical and prosthetic factors influencing perceived treatment success.

**Materials and Methods:**

A prospective study was conducted at the University Hospital of Farhat Hached, Sousse, Tunisia, from March to June 2024. Eligible patients who underwent anterior maxillary implant placement between 2018 and 2019 were included. Patients with fixed implant-supported restorations and good systemic health were eligible, while those with parafunctional habits or incomplete follow-up data were excluded. Satisfaction was assessed using a Visual Analog Scale (VAS), and OHRQoL was measured using the Oral Health Impact Profile (OHIP)-14 and Psychosocial Impact of Dental Aesthetics Questionnaire (PIDAQ) questionnaires. A total of 50 patients participated. The sample size was determined based on an expected satisfaction rate of 80%, with a 95% confidence interval and a 10% margin of error, resulting in a required minimum of 47 participants.

**Statistical Analysis:**

Data analysis was performed using SPSS version 20. Descriptive statistics summarized demographics, satisfaction scores, and OHRQoL outcomes. Inferential analyses included paired *t* -tests and Wilcoxon signed-rank tests to compare baseline and 5-year follow-up data. Spearman's correlation was used to examine relationships between implant-related factors and satisfaction or OHRQoL scores, while multivariate regression identified predictors of long-term satisfaction. A *p* -value of < 0.05 was considered statistically significant.

**Results:**

Fifty patients (mean age: 36.5 ± 5.3 years) participated, with a 98.04% implant survival rate. At 5 years, 86.2% reported high satisfaction, with a mean VAS score of 92.8%. Phonetic outcomes received a 100% satisfaction rate, while chewing comfort and stability were also highly rated (94.0 and 93.7%, respectively). Esthetic satisfaction was reported by 85.0% of patients, and 67.5% had no difficulty maintaining hygiene. The OHIP-14 median score of 9.0 indicated minimal OHRQoL impairment. PIDAQ results showed that while most patients were satisfied with esthetics, some experienced psychosocial impacts.

**Conclusion:**

Five years after anterior maxillary implant placement, patients reported high satisfaction, especially regarding phonetics, chewing comfort, and esthetics. The minimal OHRQoL impact and low PIDAQ scores highlight the positive psychosocial effects of implant restorations. These results suggest that long-term evaluation of functional and psychological outcomes should be integrated into clinical decision-making and future prospective studies.

## Introduction

Anterior maxillary edentulism presents both functional and esthetic challenges for patients and clinicians. It is well established that tooth loss can negatively impact patients' quality of life, affect mastication, speech, and self-esteem, while also influencing social interactions and psychological well-being. The prevalence of anterior maxillary tooth loss varies between populations, but studies report rates ranging from 3 to 6% in middle-aged adults, highlighting the clinical relevance of this condition. Dental implant therapy, particularly implant-supported restorations, has become the gold standard for rehabilitating these deficiencies and improving oral health-related quality of life (OHRQoL) in the long term. Numerous studies have demonstrated that implant-supported restorations significantly enhance functional and psychosocial outcomes compared to conventional prostheses. For example, Pjetursson et al 1 reported implant survival rates exceeding 95%, with marked improvements in patient-reported satisfaction and confidence. However, the success of implant treatment is not solely determined by implant survival, it also depends on patient satisfaction and the ability of the restoration to mimic the natural dentition, ensuring proper integration with both hard and soft tissues. 2 3 4 5 6

The rehabilitation process involves both surgical and prosthetic phases, each playing a critical role in achieving optimal outcomes. Implant positioning, soft tissue management, and prosthetic design must be carefully planned to achieve esthetic and functional success. Factors such as adequate keratinized tissue, biological width preservation, and three-dimensional (3D) implant positioning influence long-term stability and patient-perceived outcomes. 7 8 9 10

Beyond clinical success, patient satisfaction remains a crucial measure of treatment efficacy. Patient satisfaction can be defined as the individual's overall evaluation of the dental care received, particularly regarding functional and esthetic outcomes, while OHRQoL reflects the extent to which oral conditions affect daily activities, well-being, and social functioning. Patient-reported outcome measures provide valuable insight into perceived benefits and the overall impact on quality of life. Instruments such as the Oral Health Impact Profile (OHIP) and the Psychosocial Impact of Dental Aesthetics Questionnaire (PIDAQ) have been widely used to assess OHRQoL and esthetic perception. 11 12

While numerous studies have reported high survival rates for anterior maxillary implants, long-term data focusing specifically on patient satisfaction and OHRQoL remain limited, particularly in the context of esthetic zone rehabilitation. Moreover, few studies have comprehensively assessed the psychosocial impacts of anterior implant therapy over extended periods, representing a gap in the existing literature. Evaluating these 5 years posttreatment is essential to understanding the true benefits of implant-supported rehabilitation and guiding future improvements in clinical protocols. 13 14

The present study aims to assess patient satisfaction and OHRQoL 5 years after anterior maxillary implant placement, addressing the need for longitudinal evaluations of functional and psychosocial outcomes. We hypothesize that implant-supported restorations in the esthetic zone maintain prominent levels of patient satisfaction and minimal or no perceived impact on OHRQoL over time.

## Materials and Methods

### Study Design

This prospective, descriptive, and analytical study was conducted at the University Hospital of Farhat Hached, Sousse, Tunisia. Data collection occurred between March 2024 and June 2024, representing a 4-month period. However, this study examines patients who received implants between 2018 and 2019, thus evaluating outcomes for 5 years postimplantation.

#### Ethical Considerations

This study was conducted in compliance with ethical standards to ensure the protection of participant rights and data confidentiality. Approval was obtained from the Institutional Ethics and Research Committee of the University Hospital Farhat Hached in Sousse, Tunisia (Reference Number: IORG 0007567 ERC04112024). Participant anonymity was strictly maintained, and all personal data were handled securely throughout the study. Before enrollment, written informed consent was obtained from all participants prior to inclusion in the study. Furthermore, no conflicts of interest were identified in relation to this study.

### Study Population

This exhaustive study aimed to include all eligible patients who received anterior maxillary implants at the University Hospital of Farhat Hached, Sousse, Tunisia, between 2018 and 2019. Based on hospital records, an estimated 52 patients received anterior maxillary implants during this period.

Of the 52 eligible patients identified, 38 met all inclusion criteria and completed the 5-year follow-up. Due to the exhaustive nature of the sampling process, no formal power analysis was conducted.

The final sample size will be determined by the number of patients who meet the following inclusion and exclusion criteria.

### Inclusion Criteria

Patients treated at the University Hospital of Farhat Hached, Sousse, who received at least one anterior maxillary implant (canine to canine) between 2018 and 2019.

Patients who completed prosthetic rehabilitation with single crowns or fixed implant-supported restorations.

Patients in good systemic health or with controlled medical conditions that do not interfere with implant therapy.

Patients who provided written informed consent to participate in the study.

### Exclusion Criteria

Patients who underwent additional surgical interventions (e.g., soft tissue grafting, implant replacement) after the initial implant placement.

Patients with severe parafunctional habits such as bruxism, which may affect implant longevity.

Patients with incomplete medical or prosthetic records.

Patients who did not attend the 5-year follow-up appointment.

### Implant and Prosthetic Protocol

All patients were treated following a standardized clinical protocol, while this protocol aimed for consistency, variations may have occurred based on individual patient needs.

#### Surgical Phase

- Implant placement was performed according to the 3D positioning principles, ensuring adequate space for soft tissue adaptation and prosthetic emergence profile.
- Immediate or delayed placement was selected based on bone volume, soft tissue condition, and primary stability.
- Bone grafting was performed when necessary to compensate for hard tissue deficiencies.

#### Prosthetic Phase

- After a healing period of 3 to 6 months, the final prosthetic restorations were delivered.
- Prosthetic materials: All patients received ceramic or zirconia crowns for optimal esthetics and function.
- Abutment selection was based on soft tissue thickness, implant position, and biologic width considerations.

### Data Collection and Follow-Up

- Patients were contacted and scheduled for a follow-up visit at the University Hospital of Farhat Hached, Sousse.
- All questionnaires (Visual Analog Scale [VAS], OHIP-14, PIDAQ) were administered by a single trained examiner who followed a standardized protocol to minimize response bias by maintaining neutral and consistent interactions with participants.
- Clinical evaluations were conducted by a prosthodontist to assess the condition of the implant-supported restorations and surrounding tissues.

### Outcome Measures

- Patient Satisfaction

#### Patient Satisfaction

Patient satisfaction was assessed using a VAS, ranging from 0 (completely dissatisfied) to 10 (completely satisfied). This scale provided an overall measure of patient satisfaction with their implant restorations. To gain a more detailed evaluation, patients were also asked to respond to specific questions regarding comfort, esthetics, function, and speech. These questions were designed to assess various aspects of the patient's experience with their implant restorations:

- On a scale of 0 to 10, how satisfied are you with your anterior implant restorations overall?
- How satisfied are you with your ability to speak clearly with your implant restorations? (0 = Very dissatisfied, 10 = Very satisfied)
- Have you experienced any difficulty with chewing or biting after receiving the implant restorations? (0 = No difficulty, 10 = Severe difficulty)
- How stable do you feel your implant restorations are? (0 = Not stable at all, 10 = Very stable)
- How difficult is it to maintain hygiene around your implants? (0 = Very difficult, 10 = Very easy)
- How satisfied are you with the appearance of your implant restorations? (0 = Very dissatisfied, 10 = Very satisfied)

#### Oral Health-Related Quality of Life

In this study, the OHRQoL was assessed using the OHIP-14, a validated tool designed to measure the psychosocial and functional impact of oral health on daily life. The OHIP-14 is a shorter version of the original OHIP-49 and is widely used to evaluate the extent to which oral health problems influence a patient's well-being, including their functional abilities, emotional state, and social participation.

The OHIP-14 consists of 14 items, each focusing on a specific aspect of the patient's experience with oral health, ranging from functional limitations and discomfort to social participation. The questionnaire evaluates the frequency with which patients have experienced difficulties or negative effects in the past month, reflecting how oral health conditions, such as implant therapy, may influence their quality of life. 15

The full OHIP-14 evaluates the following domains:

1. Functional limitations: Impact on basic functions such as speaking, eating, and chewing.
2. Physical pain: Experiences of pain or discomfort related to oral health.
3. Psychological discomfort: Emotional distress linked to the appearance or function of the oral structures.
4. Physical disability: Limitations caused by oral health issues in physical activities (e.g., difficulty eating or speaking).
5. Psychological disability: Impacts on a patient's emotional state or mental health, caused by oral health problems.
6. Social disability: Restrictions in social interactions and participation in society due to oral health issues.
7. Handicap: The overall burden oral health problems impose on a patient's quality of life.

Each item in the OHIP-14 is rated on a Likert scale ranging from 1 (never) to 5 (very often). The higher the score, the greater the perceived impact of oral health on the patient's quality of life. The total OHIP-14 score ranges from 0 (no impact on quality of life) to 56 (severe impact), with higher scores indicating more significant impairment in daily life.

The responses to the OHIP-14 were categorized and analyzed to identify patterns in the patients' experiences, with the goal of understanding how implant restorations impact both their physical well-being and emotional health. Higher total scores indicated more frequent and intense effects on the patients' daily lives, while lower scores signified a minimal impact from the dental condition being treated.

- Psychosocial impact and esthetic concerns: PIDAQ scores

To evaluate the psychosocial consequences of dental esthetics following implant therapy, the PIDAQ was used. 16

This questionnaire is specifically designed to assess the impact of dental esthetics on a patient's self-esteem, social interactions, and psychological well-being. It consists of 23 questions; each answered on a Likert scale ranging from 0 (no impact) to 4 (maximum impact). The total PIDAQ score is calculated by adding the scores from four subscales, providing a comprehensive evaluation of the psychosocial impact of dental esthetics.

The PIDAQ subscales include:

- Dental self-confidence: Assesses the patient's confidence in their appearance, with a higher score indicating lower self-confidence regarding dental esthetics.
- Psychological impact: Measures the emotional distress caused by dental appearance, with higher scores indicating greater distress.
- Social impact: Reflects the impact of dental esthetics on the patient's social interactions and relationships.
- Esthetic concerns: Focuses on the patient's level of concern about the appearance of their teeth.

The total PIDAQ score ranges from 0 (no impact) to 52 (maximum impact), with higher scores indicating a greater negative impact of dental esthetics on the patient's psychosocial well-being.

### Statistical Analysis

Data analysis was performed using SPSS version 20 (IBM Corp., Armonk, New York, United States). Appropriate statistical methods were employed to ensure reliable and meaningful results. Descriptive statistics, including means, standard deviations (SDs), and frequency distributions, were used to summarize patient demographics, satisfaction scores, and OHRQoL outcomes. To compare patient-reported outcomes between baseline and the 5-year follow-up, paired *t* -tests were applied for normally distributed data, while Wilcoxon signed-rank tests were used for nonnormally distributed variables. To explore potential relationships between implant-related factors (such as implant positioning and soft tissue thickness) and patient satisfaction or OHRQoL scores, Spearman's correlation analysis was performed. Additionally, a multivariate regression analysis was conducted to identify predictors of long-term patient satisfaction, considering clinical and prosthetic variables. A *p* -value of less than 0.05 was considered statistically significant for all tests.

**Fig. 1:**
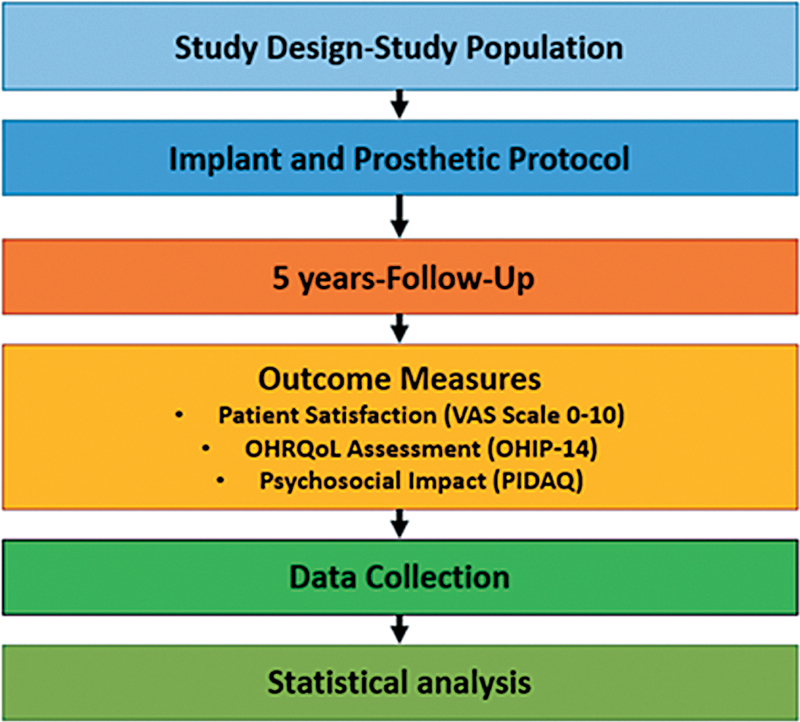
Flow diagram.

A flow diagram is provided for ease of understanding ( Fig. 1 ).

## Results

### Demographic Characteristics

**Fig. 2:**
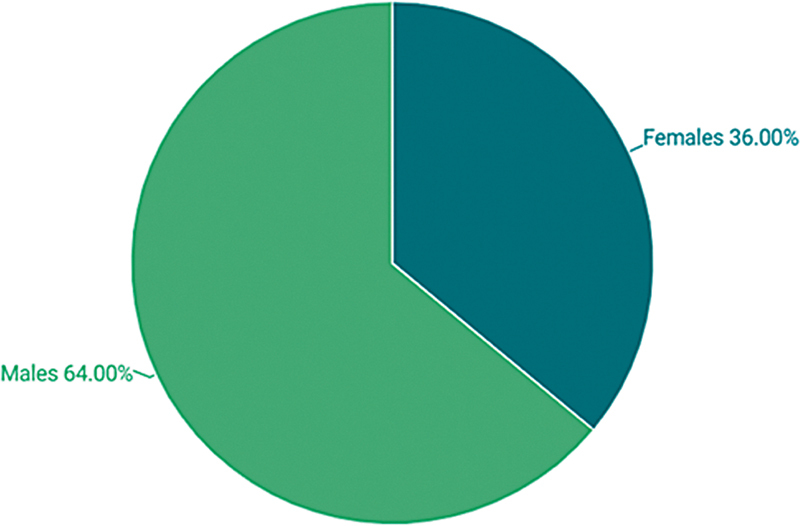
Population distribution by gender: female vs. male.

The study enrolled a total of 50 patients, with a median age of 36 years (mean: 36.5 years, SD: ± 5.3 years). The cohort included 18 females and 32 males, yielding a male-to-female ratio of approximately 1.78 ( Fig. 2 ).

#### Implant Characteristics and Outcomes

**Fig. 3:**
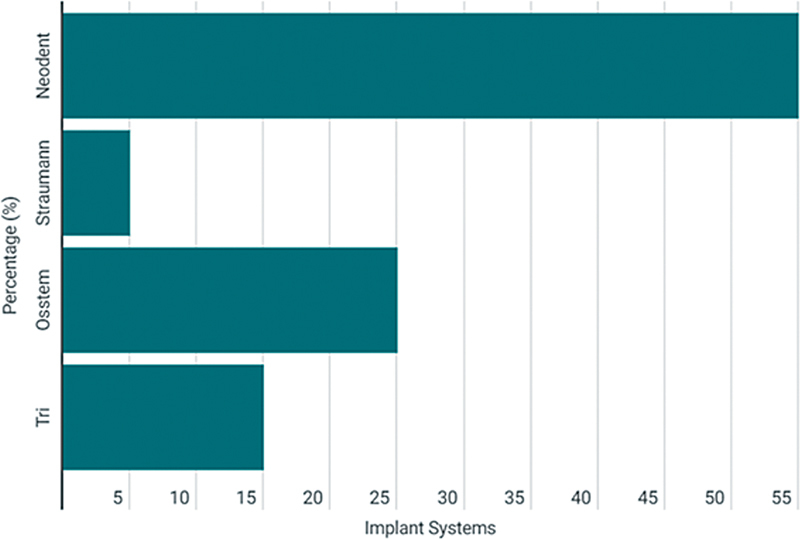
Implant systems used for patients in our study.

In total, 102 implants were placed, predominantly in the anterior maxillary region, using Neodent (55%), Straumann (5%), Osstem (25%), and Tri (15%) implant systems. During the follow-up period, two implants failed approximately 16 weeks after placement, which was discovered during the second surgical stage. There was no apparent radiographic evidence or clear reason for the failure, but the failed implants were curetted and scheduled for reimplantation 6 weeks later. After this intervention, the treatment plan proceeded without further complications. The cumulative implant survival rate for the study was 98.04% (mean: 98.5%, SD: ± 1.3%) ( Fig. 3 ).

Regarding patient profiles and treatment details:

- 28% of the participants were smokers, a factor analyzed for potential impact on implant survival, but no statistically significant difference was found ( *p* = 0.412).
- 55% received fixed single crowns, while 45% were treated with implant-supported bridges.
- 62% of patients received immediate implants following tooth extraction, while 38% had delayed implants. However, survival rates between immediate and delayed placement showed no significant difference ( *p* = 0.658).

**Fig. 4:**
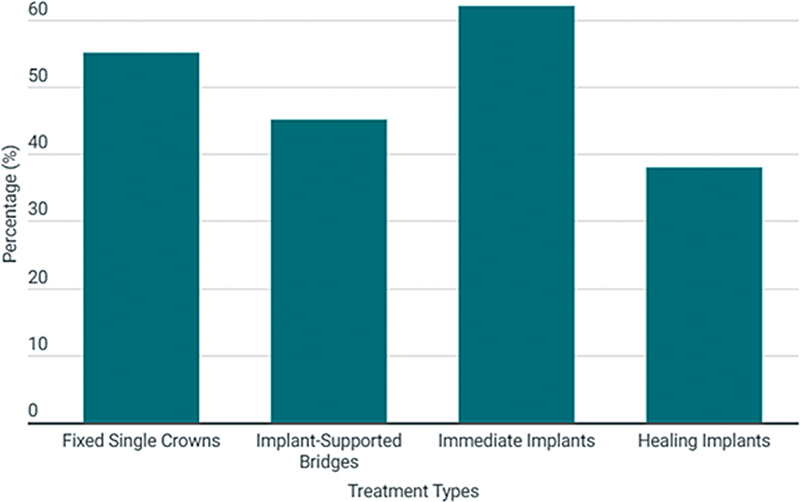
Treatment types used for patients.

Esthetic considerations were prioritized for all patients, and immediate provisional prostheses were provided using PMMA (polymethylmethacrylate) crowns. These provisional restorations ensured both functional and esthetic outcomes during the healing phase, helping to maintain optimal tissue contour and appearance ( Fig. 4 ).

The follow-up time for the study was 5 years ± 6 months (mean: 5.1 years, SD: ± 0.4 years), providing a comprehensive long-term assessment of implant success and patient satisfaction.

#### Patient Satisfaction

**Fig. 5:**
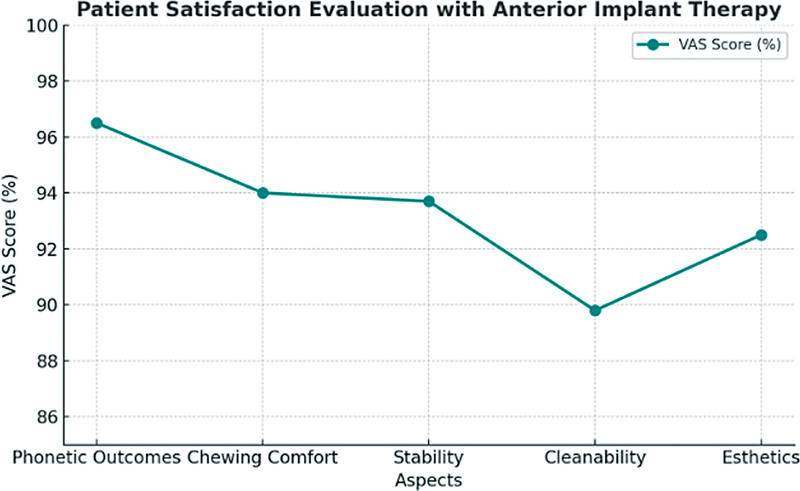
Patient satisfaction evaluation with anterior implant therapy across various outcomes. This line graph presents the mean Visual Analog Scale (VAS) satisfaction scores (in %) reported by patients for five specific aspects of anterior implant therapy: phonetic outcomes, chewing comfort, prosthetic stability, cleanability, and esthetics. The *y* -axis indicates the VAS score as a percentage, while the *x* -axis represents the different evaluated domains of implant therapy satisfaction. Higher scores reflect greater levels of patient satisfaction. Notably, phonetic outcomes achieved the highest satisfaction, whereas cleanability showed comparatively lower satisfaction.

Patient satisfaction was assessed using a VAS ranging from 0 to 100 ( Fig. 5 ). Regarding overall satisfaction, most patients (86.2%) reported being strongly satisfied with their implant therapy, while 12.4% indicated they were mostly satisfied, and only one patient (1.4%) expressed mild dissatisfaction. The mean VAS score for overall satisfaction was 92.8% (SD ± 9.2), with a median of 95.0% and a range of 55.0 to 100%. Statistical analysis revealed no significant differences in overall satisfaction when comparing genders ( *p* = 0.327) or smoking status ( *p* = 0.481).

In terms of phonetic outcomes, all patients (100%) reported being strongly satisfied, with a mean VAS score of 96.5% (SD ± 6.1; 95% confidence interval [CI]: 94.7–98.3%). The implant system used did not significantly influence phonetic satisfaction ( *p* = 0.712).

For chewing comfort, 90.5% of patients were strongly satisfied, 8.2% experienced minor restrictions, and 1.3% reported minor difficulty. The mean VAS score for chewing comfort was 94.0% (SD ± 8.4; 95% CI: 91.5–96.5%). Interestingly, patients who received single crowns reported significantly higher satisfaction compared to those with bridges ( *p* = 0.048).

Regarding prosthetic stability, 87.1% of patients found their restorations to be “very stable,” while 11.9% indicated they were “mostly stable.” The mean VAS score for stability was 93.7% (SD ± 8.8; 95% CI: 91.1–96.3%) (ajouté). No significant differences were found between the immediate and delayed implant placement groups ( *p* = 0.561).

For cleanability, 67.5% of the patients reported no difficulty maintaining hygiene around the implants, while 30.2% stated they had “mostly no problem,” and only 2.3% experienced some cleaning difficulty. The mean VAS score was 89.8% (SD ± 11.4; 95% CI: 86.4–93.2%). Smokers reported significantly lower satisfaction regarding cleanability compared to nonsmokers ( *p* = 0.036).

With respect to esthetics, 85.0% of patients were very satisfied with the final appearance of their restorations, 13.0% were mostly satisfied, and 2.0% were less satisfied. The mean VAS score for esthetics was 92.5% (SD ± 10.9; 95% CI: 89.2–95.8%). The type of implant system had no statistically significant impact on esthetic satisfaction ( *p* = 0.774).

### Oral Health-Related Quality of Life

**Fig. 6:**
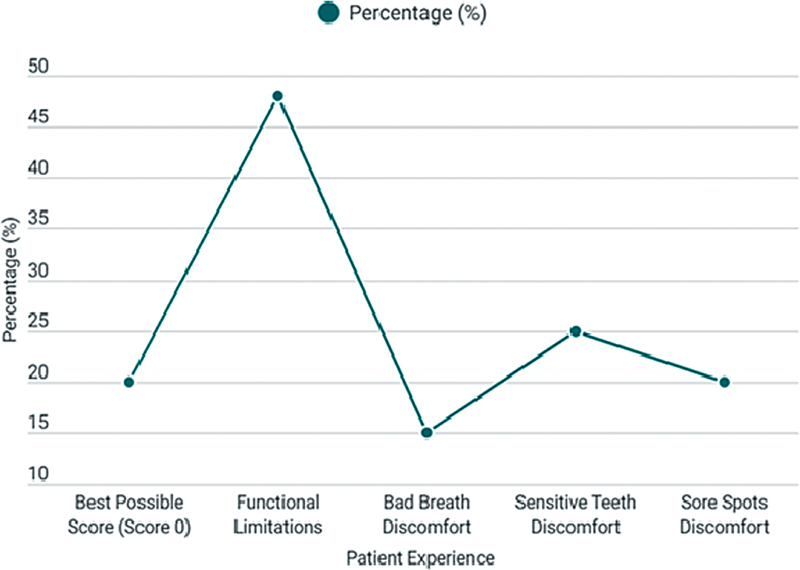
Analysis of oral health-related quality of life (OHRQoL) scores highlighting patient experiences and limitations.

The assessment of OHRQoL using the OHIP-14 scale revealed a median score of 9.0 (range 0–22), indicating minimal or no impairment in the patients' quality of life ( Fig. 6 ). Notably, 20% of the cohort ( *n* = 10) achieved a perfect OHIP-14 score of zero, suggesting no negative impact on their oral health-related well-being.

No statistically significant differences in OHIP-14 scores were observed when comparing smokers to nonsmokers ( *p* = 0.517) or between patients with single crowns versus implant-supported bridges ( *p* = 0.249). However, 48% of participants reported functional limitations, such as food impaction between teeth or beneath the prosthesis, but these issues were not significantly different between the immediate and delayed implant placement groups ( *p* = 0.621). Additionally, 15 to 25% of patients experienced minor discomforts (e.g., bad breath, sore spots), yet no significant correlation was found with the type of implant system used ( *p* = 0.783).

### Psychosocial Impact and Esthetic Concerns: PIDAQ Scores

**Fig. 7:**
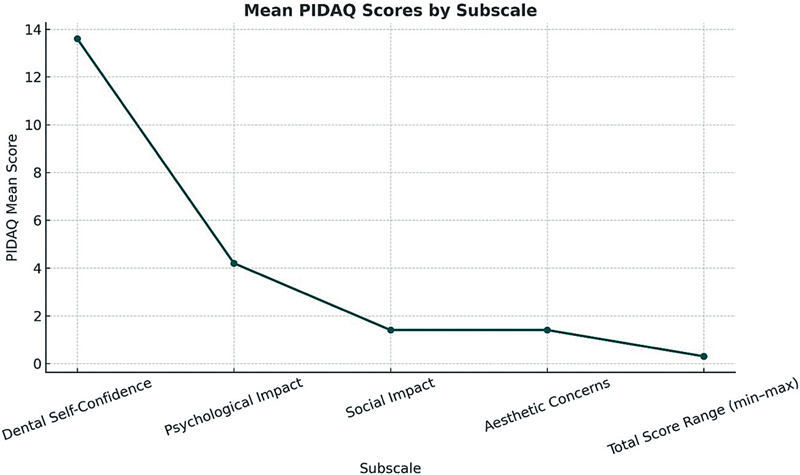
Distribution of mean scores across Psychosocial Impact of Dental Aesthetics Questionnaire (PIDAQ) subscales assessing the psychosocial and esthetic impact of dental appearance. This line graph displays the mean scores for each subscale of the PIDAQ, including dental self-confidence, psychological impact, social impact, and esthetic concerns, along with the overall score range (minimum–maximum). The results highlight the relative contribution of each domain to the participants' perception of their dental esthetics.

Alongside the OHIP-14, the PIDAQ was used to evaluate the psychological aspects of dental esthetics. The mean PIDAQ score for the cohort was 20.5 (SD **±** 11.3), with a median of 17.0 and a range from 0 to 52. These results suggest that, while the majority of patients were generally satisfied with the esthetic outcomes of their implant therapy, there was considerable variability in how they perceived the changes to their dental appearance. Some individuals, despite receiving implant-supported restorations, still reported feelings of dissatisfaction or insecurity ( Fig. 7 ).

The dental self-confidence score, which assesses patients' self-awareness and confidence in their dental appearance, had a mean of 13.9 (SD **±** 5.2) and a median of 14.0. While many patients exhibited a positive sense of dental self-awareness, the range of scores indicates that some individuals still struggled with self-confidence issues related to their dental esthetics. This highlights the need for continued psychosocial support to address these concerns and ensure comprehensive patient care.

Regarding psychological impact, the mean score was 4.6 (SD **±** 4.4), with a median of 3.0. This suggests that some patients experienced psychological discomfort associated with their oral health, although the overall impact was relatively moderate. Factors such as concerns about appearance, functionality, and the potential for complications likely contributed to this discomfort.

The social impact of dental esthetics was found to be minimal for the majority of patients, with a mean score of 1.8 (SD **±** 2.9) and a median of 0.0. This indicates that most patients did not perceive their dental esthetics to significantly affect their social interactions. However, a small proportion of patients did report more significant social impacts, highlighting that dental appearance can still influence certain individuals' social experiences.

Lastly, esthetic concerns had a mean score of 1.7 (SD **±** 1.9) and a median of 1.0, reflecting that most patients were not overly concerned with the esthetic appearance of their restorations. A small percentage of patients expressed strong concerns, but these concerns were not widespread, suggesting that the majority of patients were satisfied with the cosmetic outcomes of their implant-supported restorations.

## Discussion

Anterior implant therapy, particularly in the maxillary region, presents a unique set of challenges, as it requires a delicate balance between functionality, esthetics, and patient satisfaction. Restoring teeth in this area involves not only technical expertise but also a deep understanding of patient expectations and psychological impacts. 17

This study aimed to evaluate patient satisfaction following anterior implant therapy, focusing on functional, esthetic, and psychosocial outcomes. With a sample of 50 patients averaging 36.5 years old, the study's findings provide significant insights into the performance of anterior implant restorations. The high levels of satisfaction observed align with existing research, reaffirming the positive clinical outcomes of implants in the anterior maxilla.

The results revealed that 86.2% of patients were very satisfied with their treatment, which is consistent with findings by Luo et al and Wang et al, who reported similar satisfaction levels. 4 18 Factors such as phonetic performance, chewing comfort, and stability were identified as key contributors to this satisfaction. Phonetic performance was particularly noteworthy, with all patients (100%) expressing satisfaction, highlighting its critical role in patient contentment. This finding is in agreement with Zhang et al, who also identified phonetics as a major factor influencing satisfaction postimplantation. 19 Additionally, 90.5% of patients reported satisfaction with chewing comfort, a result consistent with Wang et al, who emphasized the importance of chewing performance in overall satisfaction with implants. 4

Implant stability also proved to be a significant factor, with 87.1% of patients reporting stable implants, supporting the conclusions of Gonçalves et al, who identified implant stability as essential for long-term success and patient satisfaction. 20 Regarding OHRQoL, as measured by the OHIP-14, most patients experienced minimal disruption to daily activities. The median score of 9.0 suggests that half of the patients reported little to no impairment in their quality of life. Moreover, 20% of patients achieved the best possible score of zero, indicating that their implants had no negative impact on their well-being. These findings underscore the positive effects anterior implants can have on a patient's overall quality of life.

Despite these positive outcomes, some functional limitations were noted. Nearly half of the patients reported issues such as food trapping or discomfort associated with the restoration. These findings are consistent with those of Kim et al, who also identified functional complications with implant-supported restorations. 21 Addressing these concerns in future studies and clinical practice is crucial to align functional outcomes with patient expectations. Moreover, such discomfort may have clinical implications for maintenance protocols and prosthetic design, warranting further research to minimize plaque accumulation and improve patient-perceived hygiene ease.

The psychosocial impact, as measured by the PIDAQ score, revealed that while the majority of patients were satisfied with their implants, some expressed dissatisfaction with their dental esthetics. This aligns with research by Kim et al, who found that despite favorable clinical outcomes, some patients still experienced psychological discomfort related to their appearance. 21 Esthetics remain a key component of the patient's experience, and treatment planning should take this into account. Social impact was minimal for most patients, reflecting the general observation that dental esthetics have limited effects on social interactions. However, a small group of patients reported significant social impacts, emphasizing that dental appearance can still affect social experiences for certain individuals. There are some limitations to this study that should be considered. The small sample size and the single-center design may limit the generalizability of the findings to broader populations or different clinical settings. Additionally, the reliance on self-reported measures such as VAS, OHIP-14, and PIDAQ introduces the possibility of response bias, as patient perceptions may be influenced by personal expectations or the desire to please the care team. Moreover, the absence of a control group prevents direct comparisons with other prosthetic or implant therapies, which could have strengthened the interpretation of the outcomes. To strengthen the validity of these findings, future studies should include larger, more diverse samples, and ideally adopt a multicentric and controlled study design.

Incorporating advanced digital technologies, such as 3D facial scanning and dynamic occlusion analysis, holds significant promise for enhancing the functional and esthetic outcomes of anterior implant therapy. These tools allow for more precise prosthetic planning, improved soft tissue management, and better visualization of patient-specific anatomical structures, thereby reducing the risk of esthetic or functional mismatches. Furthermore, such technologies facilitate patient–clinician communication by enabling virtual simulations of the anticipated results, which can improve patient understanding, involvement, and satisfaction with the treatment process. Future research should explore the clinical impact and patient perception of these digital approaches to optimize their integration into routine practice.

For clinicians, this study offers valuable insights into the positive aspects of anterior implants, particularly in terms of phonetic performance, chewing comfort, and implant stability. Understanding these factors can help refine treatment approaches and improve patient outcomes. While some limitations, such as functional discomfort and esthetic concerns, were noted, these challenges can be addressed through more personalized care strategies in clinical practice. Furthermore, the role of meticulous treatment planning should be emphasized. The integration of modern tools like facial scans and dynamic occlusion using 3D imaging can significantly enhance both functional and esthetic outcomes. These technologies allow for a more accurate and personalized approach, improving communication between clinicians and patients, and increasing the likelihood of long-term success. 22 23

For future research, it would be beneficial to conduct longitudinal studies assessing the long-term functional and esthetic outcomes of anterior implant restorations, along with the evolution of patient satisfaction over extended periods. Particularly as complications such as peri-implantitis or prosthetic wear are more likely to manifest beyond the 5-year follow-up period. Comparative studies of different implant systems, such as Neodent, Straumann, Osstem, and Tri, would provide valuable insights into the effectiveness of various systems for anterior implants, helping clinicians make better-informed decisions. Additionally, further investigation into the role of digital tools, such as 3D scans and dynamic occlusion planning, would shed light on how these technologies can improve patient satisfaction, clinical outcomes, and long-term treatment success. These studies would ultimately contribute to refining implant therapies, ensuring better results for patients and more effective treatment planning for clinicians.

## Conclusion

This study demonstrates the high levels of satisfaction among patients receiving anterior implant therapy, particularly in the maxillary region, highlighting the importance of functional, esthetic, and psychosocial considerations in treatment planning. The significant positive impact on phonetic performance, chewing comfort, implant stability, and quality of life reinforces the clinical success of anterior implants. However, some functional limitations, such as food trapping and discomfort, as well as concerns over esthetics, point to areas for further improvement.

To address these challenges, clinicians are advised to adopt personalized, patient-centered treatment plans that prioritize individual needs, preferences, and expectations. This includes careful patient selection, tailored prosthetic design to minimize functional limitations, and the integration of advanced digital technologies, such as facial scanning and 3D dynamic occlusion analysis, to optimize both functional and esthetic outcomes while enhancing patient involvement in decision-making.

Nevertheless, this study is subject to limitations, notably its small sample size and single-center design, which may restrict the generalizability of the findings.

Future research should include multicentric studies involving larger and more diverse populations, as well as longitudinal follow-up exceeding 5 years, to capture late-onset complications such as peri-implantitis and to validate these findings across various clinical contexts.
